# Short and long-term differences in anthropometric characteristics and physical performance between male rugby players that became professional or remained amateur

**DOI:** 10.1016/j.jesf.2021.01.002

**Published:** 2021-01-31

**Authors:** Michael J. Hamlin, Richard W. Deuchrass, Catherine E. Elliot, Nuttaset Manimmanakorn

**Affiliations:** a Department of Tourism, Sport and Society, Lincoln University, Christchurch, New Zealand; b Faculty of Health and Environmental Science, Sports Performance Research Institute New Zealand (SPRINZ), Auckland University of Technology, Auckland, New Zealand; c Recreation Centre, Lincoln University, Christchurch, New Zealand; d Department of Rehabilitation Medicine, Faculty of Medicine, Khon Kaen University, Khon Kaen Thailand

**Keywords:** Body composition, Developmental players, Rugby union, Speed, Strength, Yo-Yo intermittent recovery Test

## Abstract

**Objective:**

The objective of this study was to investigate which anthropometric and physical performance variables characterised players that advanced to professional teams (professionals) and how these variables changed over time, compared to those that did not secure professional contracts (i.e. remained amateurs).

**Methods:**

Differences in anthropometry, strength, speed, power and intermittent running ability in 83 male rugby players collected between 2015 and 2019 were determined using repeated measures analysis.

**Results:**

When arriving for the first year of the program, forwards that went on to become professional players were older (0.4 ± 0.3 yr, mean ± 95% CI, p = 0.004), heavier (4.6 ± 2.5 kg, p < 0.001) and stronger (range 6.2–16.4%) than forwards that remained amateur. Professional forwards were also slower at sprinting (range −2.7–2.9%, p < 0.001) and had lower Yo-Yo IRT L1 (−10.8%, p = 0.03). When first arrived on the program, professional backs were taller (3.5 ± 1.8 cm, p < 0.001), heavier (4.6 ± 2.4 kg, p < 0.001) and faster over 20 m (−1.9 ± 1.7%, p = 0.03) and 30 m (−1.7 ± 1.6%, p = 0.04) compared to amateurs. Compared to amateurs, professionals had a smaller increase in body mass (−4.2 ± 2.0%, p < 0.001) and greater improvement in sprinting (3.7, 2.8, 2.8% over 10, 20 and 30-m, respectively) and Yo-Yo IRT L1 (14.7 ± 11.0%, p = 0.05) over 3 years training.

**Conclusion:**

Characteristics that are likely to assist players in becoming professionals include being older, heavier, taller and stronger.

## Introduction

Rugby union is a field-based team sport that requires players to endure a large number of high-impact collisions along with numerous maximal sprints, high speed running and other static and dynamic exertions.^1^^,^^2^ Since rugby union turned professional in 1995, research has sought to understand, among other things, what movement and performance characteristics are required of the modern rugby union player to reach professional status.^3^ Results from such research has informed strength and conditioning staff on position-specific characteristics required of rugby players including speed, strength, power, aerobic ability and anthropometry. For example, forwards are mainly involved in situations that require greater body mass, strength and power like tackling, scrummaging and mauling.^4^^,^^5^ On the other hand, backs are involved in high-speed running, evasion, and movements that require agility and therefore require a relatively lower body mass, but high levels of power and speed.^2^^,^^6^

The aim of any training programme is to efficiently and effectively improve player’s performance in a sports-specific manner without incurring illness or injury. By increasing the player’s performance ability, many professional training programmes (i.e. Institut National du Football, for footballers or the International Rugby Academy of New Zealand, for rugby union players) hope to progress the player along the performance pathway from amateur to professional athlete. To help with program prescription, players complete regular physical assessments which tend to be used in the short-term to evaluate mesocycles or short conditioning phases. However few studies on rugby union specifically have documented the long-term anthropometrical and performance changes such developmental programmes achieve. Identifying anthropometrical and physical performance characteristics that can distinguish between players of different abilities in different positions are important for the best training regimens, performance outputs, for recruitment, and for player development.^7^^,^^8^ For example, a number of studies have shown that players with superior speed, strength, aerobic endurance and repeated sprint ability have better chances of being selected onto a professional team.^9^, ^10^, ^11^ Similarly in rugby league, Till and colleagues, in a number of retrospective studies on junior rugby league players, found significant differences in anthropometric and performance variables in 13–15 year olds that subsequently went on to gain professional contracts, compared to others that remained amateur.^12^, ^13^, ^14^

While research exists on the differences between professional and amateur rugby union athletes, this research is now 7 years old, and with the continual evolution of the game, along with changes in the physical demands required of its athletes,^15^ these research findings may be outdated. In addition, this previous research was conducted on players already at the professional level.^16^ Consequently, there is a need for a study to monitor the long-term anthropometric and performance measures including strength, power, speed and aerobic endurance in developmental rugby players that at the time of testing had not reached the professional level. The usefulness of these anthropometric and performance measures in the development of a rugby player from amateur to professional can then be elucidated and used to guide training of such players.

Therefore, the purpose of this study was to investigate which anthropometric and physical performance variables characterised players that went on to be selected for professional teams and how these variables changed over 3 consecutive years before selection was made.

## Methods

This retrospective longitudinal study used a commercially available software system (Health and Sport Technologies, Ltd., trading as Metriﬁt, Millgrange, Greenore,Co., Louth, Ireland) to collect testing data on players during their time at university. The data was uploaded into the Metriﬁt system by the strength and conditioning team throughout the player’s academic year. The players were separated by general playing positions (forwards and backs) to establish differences between players in different positions and those players that went on to gain professional contracts and those players that did not (professional and amateur). The variables used are consistent with those previously used and included stature, body mass, skinfold thickness, muscular strength and power, sprint speed (via split times) and intermittent running ability.

### Participants

Anthropometric and performance measures of 83 male rugby players (mean ± SD for age = 18.9 ± 1.3 yr, range = 17.6–26.8 yr) during their time at university between 2015 and 2019 were recorded. Players were involved in a university sport scholarship program where they received nutritional, psychological, and medical advice along with individualized strength and conditioning training. All participants were young rugby players selected from age-group provincial or national representative honours (high level amateur leagues). The study was approved by the Lincoln University Human Ethics Committee (Approval Reference No. 2018–01). All subjects were informed of the benefits and risks of the investigation prior to signing an institutionally approved informed consent document to participate in the study. The dataset incorporates measures from February through to October for each year, therefore includes the major competition season for rugby players in the southern hemisphere.

### Training

Individualized training programs were developed by the strength and conditioning staﬀ at the university for each player, depending on the player’s strengths and weaknesses, playing position, time of year (in-season vs. out of season) and injury status. In most weeks, players would have at least three training sessions, one sport-speciﬁc skills session, 2 rugby club trainings and one practice game or competition (approximately 9 h) per week.

### Testing

Performance testing was completed by the same strength and conditioning trainers and the results were entered into the Metrifit database after each test. All testing procedures were standardised using each specific test’s documented procedures (see relevant references in this section), carried out at the same time of the day, under similar environmental conditions (e.g. strength measured in the lifting gym, Yo-Yo IRT L1 and speed measured on a suitable-sized indoor artificial turf). Familiarization trials were not required as all players were familiar with the testing protocols and had been tested numerous times previously. Similar to previous research^17^ individual tests were not systematically different between trials with a between-test variability (typical error) of <10%. Data on intermittent running ability (Yo-Yo IRT L1), anthropometry, upper and lower body strength, speed and power from players from the start of 2015 to the end of 2019 was downloaded from the database. For each year, players were tested approximately every 2–3 months from March to October.

### Anthropometry

Body mass, reported in kg (to 1 decimal point) was measured on calibrated scales (Seca, model 762, Germany) with the players shoes and socks removed and in light training clothing. The sum of 8 skinfolds (bicep, triceps, subscapular, abdominal, supraspinale, iliac crest, front thigh and medial calf) were taken using International Society for the Advancement of Kinanthropometry (ISAK) guidelines, by an ISAK-qualified level 3 practitioner.^18^ Skinfold thickness showed adequate between-test reliability with a typical error of 11.0%.

### Strength and power

On the first testing occasion in the participants first year of the program, 1RM was estimated from a number of resistance training exercises using a 5–10 repetition maximum protocol and the formula of Brzycki (1993)^19^ which has since been shown to have good validity in predicting 1RM (r^2^ = 0.99).^20^ Thereafter, a true 1RM was measured. The strength exercises included deadlift, back squat, bench press, chin-up and prone row. The power clean was used to indicate lower body power. All lifting attempts were observed by qualified and experienced strength and conditioning trainers and only lifts using correct technique were recorded.

During the bench press, player’s used a self-selected hand position and their feet remained in contact with the floor while the gluteus maximus and lower back remained in contact with the bench throughout the lift. During the lift, the bar was lowered to the chest (with elbows at approximately 90° with no bouncing off the chest) and returned to the start position where elbows were fully extended but not locked. The back squat required the player to descend in a controlled manner until the top of the thighs were at least parallel with the floor before returning to the standing position. The deadlift started with the weights resting on the platform and the participants were instructed to lift the barbell while maintaining a neutral, straight back and to extend their knees and hip in one movement (to avoid a straight-leg deadlift technique). The lift was completed when the hip was fully extended (the angle between the trunk and the thigh was approximately 180°). For the prone row, each player was instructed to lie on the prone row, high bench, face down, keeping their head, chest, and legs flat for the entire lift. The subjects then gripped the bar with a pronated self-selected hand grip. The players then removed the bar from the bench supports and let it hang freely. When the bar was motionless, the players raised the bar toward their chest until it came in contact with the high prone row bench. When performing the chin-ups, a reverse underhand grip (palms facing toward face) was used. Players were instructed to start from a stationary position with arms fully extended and complete a repetition with the chin moving over the bar. The power clean required the player to set up in a crouched position over the bar on the floor with fully extended arms. From this position, the player was instructed to thrust upward in a triple extension movement, pulling the barbell upward into the catch position on the front of the shoulders with elbows forward.^21^ The coefficients of variation (CV) for similar strength testing protocols within professional rugby union players have been shown to be approximately 4.5%.^22^ Testing occurred on a de-loading week so that the players were coming into the test relatively un-fatigued. During this week, squat testing was completed on Monday with bench press, prone row and chin up on Wednesday and deadlift and power clean on Thursday. Testing commenced after a light warm-up (3 min of light walking at self-selected pace) and stretch (3 min self-selected stretches). The 1RM protocol included one set of 10 reps at a relatively light load that served as a specific warm-up, followed by a gradual increase in load until 1RM was achieved. The rate of progress in load was dependent on the player’s self-perceived capacity and ranged from 1 to 20 kg. All 1RM’s were achieved within 3–6 attempts. Rest periods between attempts was 3 min, and rest periods between exercises was at least 10 min.

### Speed

All sprints were performed in suitable footwear (e.g. gym shoes) on artificial turf in an indoor stadium. The players were instructed to sprint maximally for every repetition within the lane formed by the Smart Speed (Fusion Sport, Queensland, Australia) single-beam electronic timing gates, which was approximately 2-m wide. Players started each sprint with their foot on a line 0.3 m back from the light beam of the first timing gate, in a stationary upright position, with no rocking back or forth before starting. Each player performed 2–3 repetitions and the time to complete the total distance (10, 20 or 30 m) of each sprint was recorded, with the average of the fastest 2 times recorded for analysis. Each of the 2 efforts was performed after at least a 2-min rest from the previous repetition.

### Intermittent running ability

Intermittent running ability was estimated by the 20-m shuttle run test (Yo-Yo IRT L1, BangsboSport, Denmark) which was completed according to previously-published protocols^23^ and involved players completing 2 × 20 m shuttle runs in time to an audio signal. Players started with both feet on or behind the first 20 m line and ran towards the second 20 m line aiming to reach this line in time with the audio signal. Players then turned and ran back to the starting line, again in time with the audio signal. At the end of each 2 × 20 m shuttle there was a 10 s period where players walked or jogged around a cone (placed 5 m past the finishing line) back to the starting line again for the next shuttle. The test was concluded if, after one warning, the player failed to complete a shuttle in time, or the player removed themselves voluntarily. The Yo-Yo IRT L1 finishing shuttle number was converted to a distance and then used in the analysis. The CV for Yo-Yo IRT L1 in a similar-aged group of rugby players has been shown to be 3–6.9%.^24^

### Statistical analyses

Means and standard deviations of the variables along with differences between the means of the two groups were estimated using a mixed modeling procedure (Proc Mixed) in the Statistical Analysis System (Version 9.3, SAS Institute, Cary North Carolina, USA). We analysed the natural logarithm (and transformed back if necessary) of each measure to reduce any eﬀects in non-uniformity of error and to obtain changes in measures and errors as percentages.^25^ The ﬁxed eﬀects were test time, year, group (Professional, Amateur) and their interaction. The random eﬀects were subject and residual variance. For the first year measurement, we took the mean of the measurements taken in the first year of the program. For the 3-year change measurement, we took the mean change in the variables over 3 years. Results are presented as the standardised mean difference along with the 95% confidence interval. A Pearson correlation coefficient was calculated to investigate the association between body mass, skinfold thickness and performance variables. P values are also given for the between group comparisons for those who use traditional hypothesis testing. We used an alpha level of p ≤ 0.05 for significance in this study. This study used a convenience sample of 83 players producing up to 4 data points for each dependent variable per year over a total of 3 years. The mean typical error (as a co-efficient of variation) over all tests was body mass = 2.7%, sum of 8 skinfolds = 11.0%, 10 m = 2.5%, 20 m = 2.2%, 30 m = 2.3%, back squat 6.9%, bench press = 4.8%, chin up = 4.0%, deadlift = 5.8%, prone row = 4.8%, power clean = 7.1%, Yo-Yo IRT L1 = 10.9%.

## Results

Of the 83 athletes that we collected data on over the study period, 24 (10 forwards, 14 backs) went on the gain contracts with professional rugby teams in New Zealand and overseas.

### First year of the program

In the first year of the program, forwards that went on to become professional rugby players later in their career were older (0.4 ± 0.3 years, mean ± 95% confidence interval, p = 0.004) and heavier (4.5 ± 2.5 kg, p < 0.001) than forwards that remained amateurs, however, there was no clear difference in the sum of eight skinfolds between these two groups (11.3 ± 15.4 mm, p = 0.14). Forwards that went on to become professional players were slower than amateur forwards (0.05 ± 0.03 s, p < 0.001; 0.08 ± 0.05 s, p < 0.001; 0.12 ± 0.07 s, p < 0.001 for the 10, 20 and 30 m sprint times, respectively). Differences in muscular power measured by the power clean test between the future professional and amateur forwards was not different (−3.9 ± 9.6 kg, p = 0.43). The forwards that went on to become professional players were stronger than those that remained amateur (15.3 ± 14.2 kg, p = 0.03; 19.5 ± 9.7 kg, p < 0.001; 8.5 ± 5.9 kg, p = 0.005; 10.0 ± 5.0 kg, p = < 0.001 for the deadlift, bench press, chin up and prone row, respectively). However, forwards that went on to become professional players were worse than players that remained amateur in the Yo-Yo IRT L1 test (−157 ± 149 m, p = 0.03).

Overall, there were less differences in measurements between the backs (professional versus amateur, see Table 2). In the first year of the program, backs that went on to become professional rugby players were taller (3.5 ± 1.8 cm, p < 0.001) and heavier (4.6 ± 2.4 kg, p < 0.001) than backs that remained amateurs. In addition, in the first year of the program, backs that went on to become professional players were faster (−0.06 ± 0.05 s, p = 0.03; −0.07 ± 0.07 s, p = 0.05 for the 20 and 30 m sprint times, respectively) compared to backs that remained amateur.

**Table 1:** Weekly training schedule for rugby players.

	Mon	Tue	Wed	Thur	Fri	Sat	Sun
Morning		Speed/strength (90 min)		Speed/strength/power (60 min)			
Afternoon	Strength (60 min)		Aerobic/small-sided games (20 min) Skills (60 min)			Rugby match (90 min)	
Evening		Rugby club training (90 min)		Rugby club training (90 min)			

The strength, speed and power training sessions could be scheduled in the morning or afternoon depending on player’s university commitments.

**Table 2:** Average anthropometrical and physical performance characteristics of the players in the first year of the scholarship program that went on to become professional or amateur players.

	Forwards		Backs	
	Professional (*n* = 10)	Amateur (*n* = 36)	Professional (*n* = 14)	Amateur (*n* = 23)
Age (yr)	19.2 ± 0.8	18.8 ± 1.6^a^	18.8 ± 0.7^b^	18.6 ± 0.6
Stature (cm)	187.5 ± 8.8	186.7 ± 5.9	183.9 ± 7.3^b^	180.4 ± 4.7†^c^
Body mass (kg)	104.3 ± 5.4	99.7 ± 10.2^a^	86.9 ± 7.9^b^	82.3 ± 6.7†^c^
Skinfold thickness (mm)	105.5 ± 26.4	94.1 ± 25.6	62.9 ± 12.0^b^	65.6 ± 16.7^c^
10 m sprint (s)	1.89 ± 0.12	1.83 ± 0.07^a^	1.72 ± 0.04^b^	1.75 ± 0.05^c^
20 m sprint (s)	3.23 ± 0.15	3.14 ± 0.11^a^	2.95 ± 0.09^b^	3.00 ± 0.11†^c^
30 m sprint (s)	4.50 ± 0.23	4.37 ± 0.14^a^	4.08 ± 0.08^b^	4.16 ± 0.12†^c^
Yo-Yo IRT L1 (m)	1593 ± 413	1751 ± 343^a^	2065 ± 409^b^	1979 ± 352^c^
Deadlift (kg)	180 ± 33	165 ± 13^a^	158 ± 18^b^	149 ± 21^c^
Back Squat (kg)	158 ± 26	149 ± 24	137 ± 13^b^	135 ± 16^c^
Bench Press (kg)	129 ± 17	110 ± 14^a^	100 ± 10^b^	101 ± 18^c^
Chin-Up (kg)	137 ± 10	128 ± 10^a^	121 ± 11^b^	118 ± 9^c^
Prone Row (kg)	102 ± 8	92 ± 12^a^	88 ± 7^b^	86 ± 7^c^
Power Clean (kg)	100 ± 9	96 ± 15	84 ± 16^b^	87 ± 10^c^

Data are mean ± SD. Skinfold thickness; sum of 8 skinfolds; Yo-Yo IRT L1, Yo-Yo Intermittent Recovery Test-Level 1.

a Significant difference between professional and amateur forwards (p ≤ 0.05); † Significant difference between professional and amateur backs (p ≤ 0.05).

b Significant difference between backs and forwards in professional players (p ≤ 0.05).

c Significant difference between backs and forwards in amateur players (p ≤ 0.05).

### Differences over three years

The mean 3-year change in performance within players that went on the become professionals and those that remained amateur are given in Table 3. Both groups showed increases in body mass over the 3 years, but the body mass of players that remained amateur increased more than the players that went on to become professionals (4.2 ± 2.0%, mean 3-yearly % increase ± 95% confidence interval, p < 0.001). Some of this increased body mass in the amateur group particularly, was likely to be body fat since the sum of skinfolds also increased over the 3 years in this group (8.9 ± 6.2%, p = 0.004, Table 2). Sprinting ability in all 3 sprint tests (10, 20 and 30 m) improved over the 3 years in the players that went on to become professionals (p < 0.001, p = 0.004, p = 0.009), which was not mirrored in the amateur players (p = 0.27, p = 0.53, p = 0.80). Performance in the Yo-Yo IRT L1 test improved in the individuals that went on to become professional players (9.6 ± 10.1% over 3 years, p = 0.05), but decreased in the players that remained amateur (−5.0 ± 4.6%, p = 0.03). Improvements in performance were found in the deadlift, back squat, bench press, prone row and power clean in the forwards and back squat, bench press, chin up, prone row and power clean in the backs over the 3 years in the separate professional and amateur groups with no significant difference between groups.

**Table 3:** Average 3-yearly change (%) in anthropometrical and physical performance measures in rugby players that went on to become professional or amateur players and differences between these player groups.

	Professional (*n* = 24) (% change)	Amateur (*n* = 59) (% change)	Mean Between Group Difference and 95% confidence interval
Body mass	2.9 ± 1.7^a^	7.0 ± 3.4^b^	−4.2 ± 2.0^c^
Skinfold thickness	9.6 ± 12.0	8.9 ± 6.2^b^	−0.4 ± 12.9
10 m sprint	−4.4 ± 2.5^a^	−0.7 ± 1.5	−3.7 ± 2.8^c^
20 m sprint	−3.1 ± 2.0^a^	−0.3 ± 1.0	−2.8 ± 2.3^c^
30 m sprint	−2.7 ± 2.0^a^	0.1 ± 0.9	−2.8 ± 2.1^c^
Yo-Yo IRT L1	9.6 ± 10.1^a^	−5.0 ± 4.6^b^	14.7 ± 11.0^c^
Deadlift	10.7 ± 10.6^a^	10.1 ± 14.3	0.6 ± 16.7
Back Squat	7.2 ± 6.4^a^	9.3 ± 4.4^b^	−2.2 ± 7.0
Bench Press	9.5 ± 6.4^a^	11.9 ± 5.7^b^	−2.4 ± 7.3
Chin-Up	2.9 ± 3.9	5.6 ± 2.6^b^	−2.6 ± 4.4
Prone Row	11.3 ± 5.4^a^	13.8 ± 6.6^b^	−2.5 ± 4.8
Power Clean	13.4 ± 9.1^a^	13.6 ± 6.4^b^	−0.2 ± 9.1

Data are mean percent changes of all players in each group over 3 years ± 95% confidence interval. Skinfold thickness; sum of 8 skinfolds; Yo-Yo IRT L1, Yo-Yo Intermittent Recovery Test-Level 1.

a Significant differences within professional group (p ≤ 0.05).

b Significant differences within amateur group (p ≤ 0.05).

c Significant differences between professional and amateur players (p ≤ 0.05).

Overall there was little difference between the two groups in terms of the smallest worthwhile change for each test (Table 4). In most cases a change in performance of 1–3% would be sufficient to meet the threshold for a substantially practical change, except for the sum of 8 skinfolds where a change of 5–6% would be required.

**Table 4:** The smallest worthwhile effects in anthropometrical and physical performance measures in players that went on to become professional or remained amateurs.

	Professional (*n* = 24)	Amateur (*n* = 59)
Body mass	2.3% (1.3–3.0)	2.2% (1.6–2.6)
Skinfold thickness	6.1% (2.1–8.4)	5.6% (4.1–6.8)
10 m sprint	1.2% (0.6–1.6)	0.6% (0.4–0.8)
20 m sprint	1.1% (0.5–1.4)	0.6% (0.4–0.8)
30 m sprint	1.0% (0.4–1.4)	0.6% (0.3–0.8)
Yo-Yo IRT L1	5.3% (2.4–7.2)	3.1% (2.0–4.0)
Deadlift	2.7% (1.1–3.1)	1.9% (1.0–2.5)
Back Squat	3.4% (1.7–4.6)	2.5% (1.8–3.1)
Bench Press	3.3% (1.6–4.4)	2.2% (1.5–2.7)
Chin-Up	1.9% (0.8–2.6)	1.4% (0.9–1.8)
Prone Row	1.7% (0.1–2.4)	2.0% (1.3–2.4)
Power Clean	3.3% (0.7–4.7)	2.3% (1.4–2.9)

Data are mean smallest worthwhile effect over the 3-year period (and 95% confidence interval). Skinfold thickness; sum of 8 skinfolds; Yo-Yo IRT L1, Yo-Yo Intermittent Recovery Test-Level 1.

## Discussion

Anthropometrical and physical performance measures that clearly differentiate between development rugby players that go on to become professionals or remain amateurs would be useful to sport administrators, coaches, players and physical conditioning staff. The aim of this study was to discover whether selected anthropometrical and physical performance measures might characterize such players and how these measures change over a 3 year period. We have found a number of significant differences between players who became professional compared to players that remained amateur including differences in age, body mass, sprint times and strength measures.

Similar to previous research on rugby players,^26^, ^27^, ^28^, ^29^, ^30^ forwards were generally taller and heavier than backs, whether players went on to become professional players or not. Forwards also had higher skinfold thickness, lower Yo-Yo IRT L1 performances and slower sprint times, but higher strength and power measures compared to backs. Such differences have been attributed to the different performance characteristics between forwards and backs (i.e. forwards require strength and power and need to show physical dominance in securing their own ball or stopping the opposition).^31^

Players, particularly forwards, that went on to secure professional rugby union contracts later in their careers tended to be older and heavier in their first year, than players that did not secure professional contracts. Although the forwards and backs that went on to become professionals average body mass was lighter than that reported for Super Rugby Championship players (117 and 96 kg for Super Rugby forwards and backs respectively),^30^ their body mass was significantly greater than the body mass of the amateur athletes. The body mass difference between the two groups highlights the increased requirement of size within the professional rugby game.^32^

Although the players that went on to become professionals had higher body mass compared to the amateurs, there was no significant difference in the skinfold thickness between the groups. In players that became professionals, the higher body mass was associated with longer sprint times (r = 0.61. 0.73, 0.74 for the 10, 20 and 30 m sprints, respectively) and decreased Yo-Yo IRT L1 distance (r = −0.64), but significantly greater performance in most strength and power tests (ranged from r = 0.24 to 0.65). Similar to previous research,^33^ we found body mass is clearly an important factor when considering level of performance in rugby.

As might be expected, a concentrated and specific training program over 3 years in all players resulted in some significant anthropometrical and physical changes. Because rugby requires high levels of strength and speed,^34^ there was a major emphasis on strength and power training over the 3 years (Table 1), with approximately 40% of training spent on strength and power conditioning. Typically, such training results in skeletal muscle hypertrophy^16^ which could account for the substantial increase in body mass of the 2 groups over the 3-year training period. However, the substantial increase in skinfold thickness, in the amateur players at least, indicates that some of the increase in body mass during the training period may be associated with increased fat mass.

The major differences between groups in their adaptation to training over the 3-year period was substantially improved sprinting and Yo-Yo IRT L1 ability in the players that went on to become professional compared to those that remained amateur. Having higher skinfold thickness which can reasonably indicate fat mass,^35^ may result in less power-to-weight ratio and lower sprint speed,^36^ therefore the professional players would have an advantage here. The increased mass in the amateur players would also result in lower Yo-Yo IRT L1 scores, given the number of changes in direction and the accompanying deceleration and acceleration required with such tests.^37^ In a recent study on rugby league players, Scott et al. (2017) also found that body mass and sum of 7 skinfolds were negatively correlated to intermittent running ability.^38^ During the three years of the program, players are exposed to selection processes for higher team selection and players start to comprehend whether they will progress as professional athletes or not. The gradual realization that a professional career is beyond some players may help to explain some of the changes in body composition (i.e. increased fat mass) and performance witnessed over time between the groups.^9^ Although speculative, it is our belief, in our players at least, that players in contention for professional team selection tend to train harder and with more intent than players that are not. In addition, players making selection teams have increased access to technical and tactical coaching as well as training more and playing more games over the season compared to those not in selection contention.

Improvement in strength and power over the training period was similar in both groups with no clear differences in the change scores between groups (Table 3). In most cases, improvement was in the range from 3 to 13% over the 3 years. Indeed, when looking at similar research over longer term studies, the average yearly improvement in strength and power (taken as an average yearly change in similar tests; bench press, back squat, chin-up, power clean) in the players of this study were similar to New Zealand provincial players (2.8% per year), but substantially lower than Super Rugby or International players (3.2% and 4.0% per year respectively).^16^ The greater improvements in strength and power of the Super Rugby and International players compared to the players in this study, are probably due to playing more games, training more, access to high quality nutrition and effective recovery stratergies.^39^

Regular measurement of physical performance and body composition data over time is an important aspect of monitoring athletes. Such information is useful in making decisions about the magnitude of change in these variables and thereby the success or otherwise of the training. To make inferences about practically important changes we have calculated the average (over all tests) smallest worthwhile change for each of the tests used (Table 4). Such data needs to be used in conjunction with the magnitude of the typical error of each test to identify changes of substantial practical importance for the players. For example, the typical error (as a coefficient of variation) for the Yo-Yo IRT L1 in the rugby players of this study, indicate intermittent running performance varied by approximately 10.9% from test to test when conducted 1–2 months apart. This reliability in relation to the smallest worthwhile change in Table 4 (3–5%) suggests there is too much variation in performance to identify changes of the smallest worthwhile effect and that only larger effect sizes will be noticed until measures to reduce the test-to-test variation are implemented.

A limitation to this study is the fact that a large number of variables are involved in rugby union performance and success and can therefore effect the chances of players securing professional contracts. Many of these variables (injury, skill level, attitude to training and recovery, diet, willingness to train, adaptability) have not been measured in this study, and therefore cannot be ruled out as important indicators for players achieving professional contracts. In addition, a number of the field tests used in this study have not been validated in a rugby union context and results should be viewed with caution until validation studies on these tests have been performed. Because the study has used skinfold thickness and not more accurate body composition measures the study cannot elucidate how changes in body composition might affect the results. A further limitation is the relatively small subject sample of 83 players, especially when analyzing sub-groups. Due to this small sample size, the results are not intended to be interpreted as normative for all rugby union players, and differences between players that became professional compared to those that remained amateur should be viewed with caution until further analysis on greater subject numbers is conducted. Finally, as the subject sample is from one university rugby club, the results have the potential to be biased and influenced by factors associated with this club such as coaches, strength and conditioning staff etc.

## Conclusion

Forward rugby players that go on to secure professional contracts later in their careers tend to be older, heavier and stronger, but not necessarily faster or more aerobically fit than forwards that remain amateurs. Whereas, backs that go on to become professional players tend to be taller, heavier but faster than backs that remain amateur. Players (both forwards and backs) that go on to secure professional contracts tend to improve strength and power performance over time while maintaining body fat levels. Additionally, calculating the smallest worthwhile effects from regular performance and anthropometrical tests will help coaches verify whether players are making meaningful improvements over time.

## CRediT authorship contribution statement

**Michael J. Hamlin:** Conceptualization, Methodology, Data curation, Formal analysis, Writing - original draft. **Richard W. Deuchrass:** Investigation, Writing - review & editing, Data curation. **Catherine E. Elliot:** Methodology, Writing - review & editing. **Nuttaset Manimmanakorn:** Methodology, Writing - review & editing.

## Declaration of competing interest

None.
